# Arrhythmogenic Cardiomyopathy: A Review of a Rare Case of Biventricular Phenotype

**DOI:** 10.7759/cureus.30040

**Published:** 2022-10-07

**Authors:** Henry O Aiwuyo, Gulfam Javed, Omotomilola Ataiyero, Emeka C Ibeson, Beatrice Torere, Ejiro M Umuerri, Taha El Hadj Othmane

**Affiliations:** 1 Internal Medicine, Brookdale University Hospital Medical Center, Brooklyn, USA; 2 Cardiology, East Suffolk and North Essex NHS Foundation Trust, Colchester, GBR; 3 Internal Medicine, Maimonides Medical Center, Brooklyn, USA; 4 Internal Medicine, North Mississippi Medical Center, Tupelo, USA; 5 Internal Medicine/Cardiology, Delta State University Teaching Hospital, Oghara, NGA

**Keywords:** sudden cardiac death (scd), heart failure, arrhythmogenic right ventricular cardiomyopathy (arvc/d), biventricular phenotype, arrhythmogenic right ventricular dysplasia

## Abstract

Arrhythmogenic cardiomyopathy is a rare hereditary structural heart disease, with various phenotypes, which mostly affects the right ventricle of the heart, resulting in fibrofatty replacement of the heart muscles and a proclivity to create spontaneous malignant cardiac arrhythmias that may lead to sudden death. Most previous reports were noted on young people. We report a case of its biventricular phenotype in a 61-year-old heavy truck driver who has a current medical history of diabetes mellitus and smoking and was incidentally diagnosed based on the Padua criteria after presenting to the hospital with complaints of lightheadedness and syncope. He eventually had an implantable cardioverter defibrillator, hence preventing death. We were able to correctly diagnose the case and prevent sudden cardiac death by instituting the necessary management.

## Introduction

Arrhythmogenic cardiomyopathy (ACM) characterizes a subset of structural heart disease affecting predominantly the right ventricle of the heart with a fibrofatty replacement of the heart muscles [1-5] and with a tendency to generate spontaneous malignant cardiac arrhythmias that could lead to sudden death [6,7]. While it is known to be a rare disease, there have been well-documented reports of this unique form of cardiomyopathy. Patients remain asymptomatic for most of their lives until they experience life-threatening electrical abnormalities [8].

It was first described by Guy Fontaine, a French cardiologist and electrophysiologist, in 1977 [9]. The prevalence of this disease ranges from 1:2500 to 1:5000 [6,8,10] with a male preponderance of 2.7:1 (male: female) [11]. The disease has been described as autosomal dominant with variable penetrance [12]. A rare autosomal recessive variant has been described, which is associated with woolly hair and palmoplantar keratoderma (Naxos disease) [13,14]. Genetic mutations in desmosome proteins form the basis of pathogenicity [12], and family members of patients with this condition are expected to have genetic screening for early identification and prevention of sudden death [3,12].

## Case presentation

**Figure 1:**
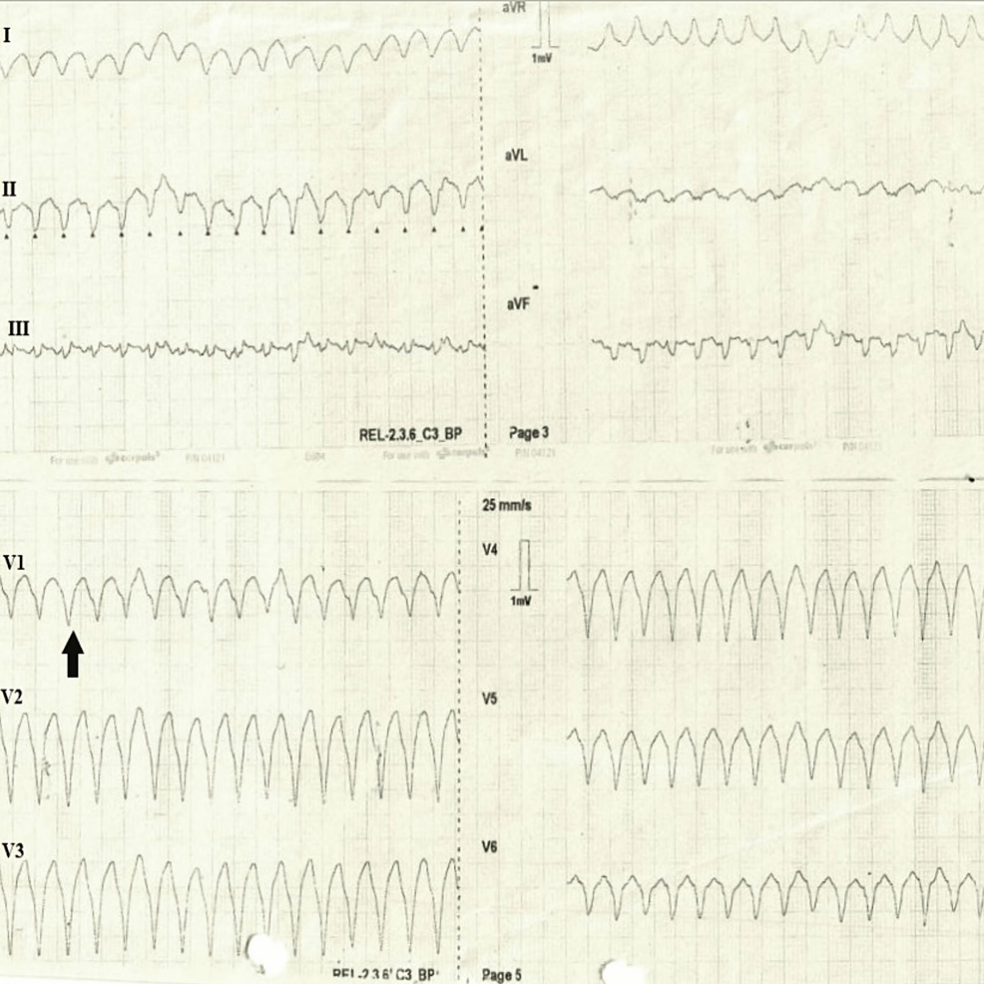
Ventricular tachycardia Black arrow shows the left bundle branch morphology

**Figure 2:**
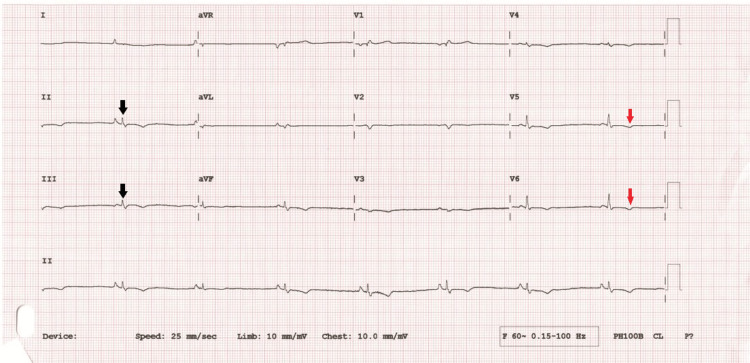
Post-cardioversion ECG Black arrow shows low limb voltage; red arrows show T-wave inversion in lateral leads

**Figure 3:**
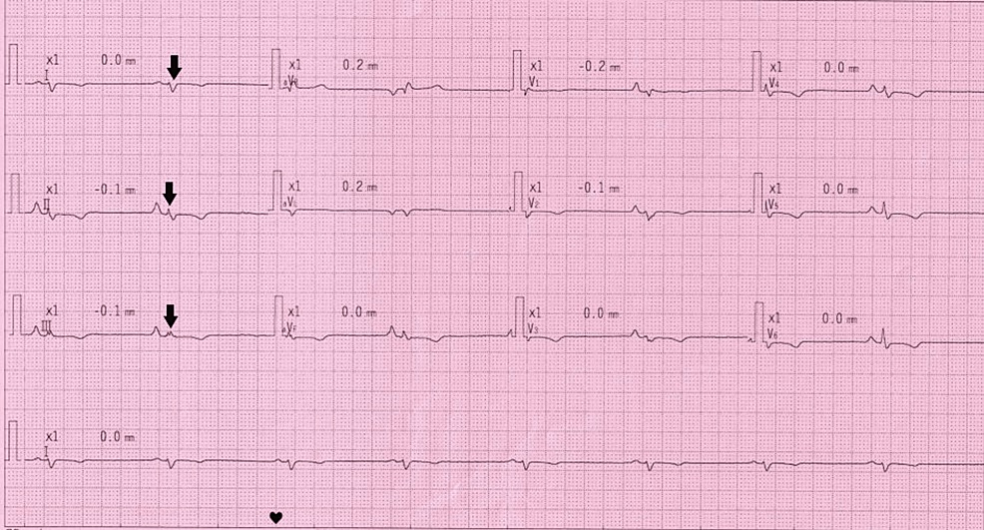
Fontaine ECG Black arrow shows right axis deviation and low-limb voltages

Our patient is a 61-year-old gentleman of western European descent who works as a heavy vehicle driver. He has had a previous episode of lightheadedness, which he did not report because he felt it was due to heat exhaustion. Then, he developed unwitnessed syncopal episodes while trying to offload a truck. He denied any history of chest pain, shortness of breath, or orthopnea. He was previously asymptomatic with a background history of type 2 diabetes mellitus, and he is a current smoker. He had no family history of any heart disease but there was a history of recurrent dizziness in his son, which was yet to be investigated, however, there was no family history of sudden death or drowning. There were no history or examination findings suggestive of postural hypotension or situational syncope, and his physical examination findings were largely unremarkable. Significant abnormalities in his blood investigations were troponin T - 134, 215 (consecutively taken six hours apart) and elevated N-terminal pro-brain natriuretic peptide (NT-Pro-BNP) of 1802. Other blood tests, including electrolytes and thyroid function tests, were essentially normal. On admission, an electrocardiogram (ECG) revealed monomorphic ventricular tachycardia (VT) with a left bundle branch block (LBBB) pattern (suggesting a right ventricular origin of the VT; Figure 1). The axis was in keeping with a right ventricular (RV) apical origin, and he had a spontaneous resolution back to sinus rhythm while in the emergency room. The ECG tracing post-VT event revealed right axis deviation and low limb voltages (Figure 2). Fontaine ECG with the right arm electrode on the manubrium, the left arm electrode over the xiphoid process, and the left leg electrode in the standard V4 position (5^th^ intercostal space, mid-clavicular line), creating F-I, F-II, and F-III leads. This transformation showed right axis deviation and low limb voltages with no epsilon waves detected (Figure 3).

**Figure 4:**
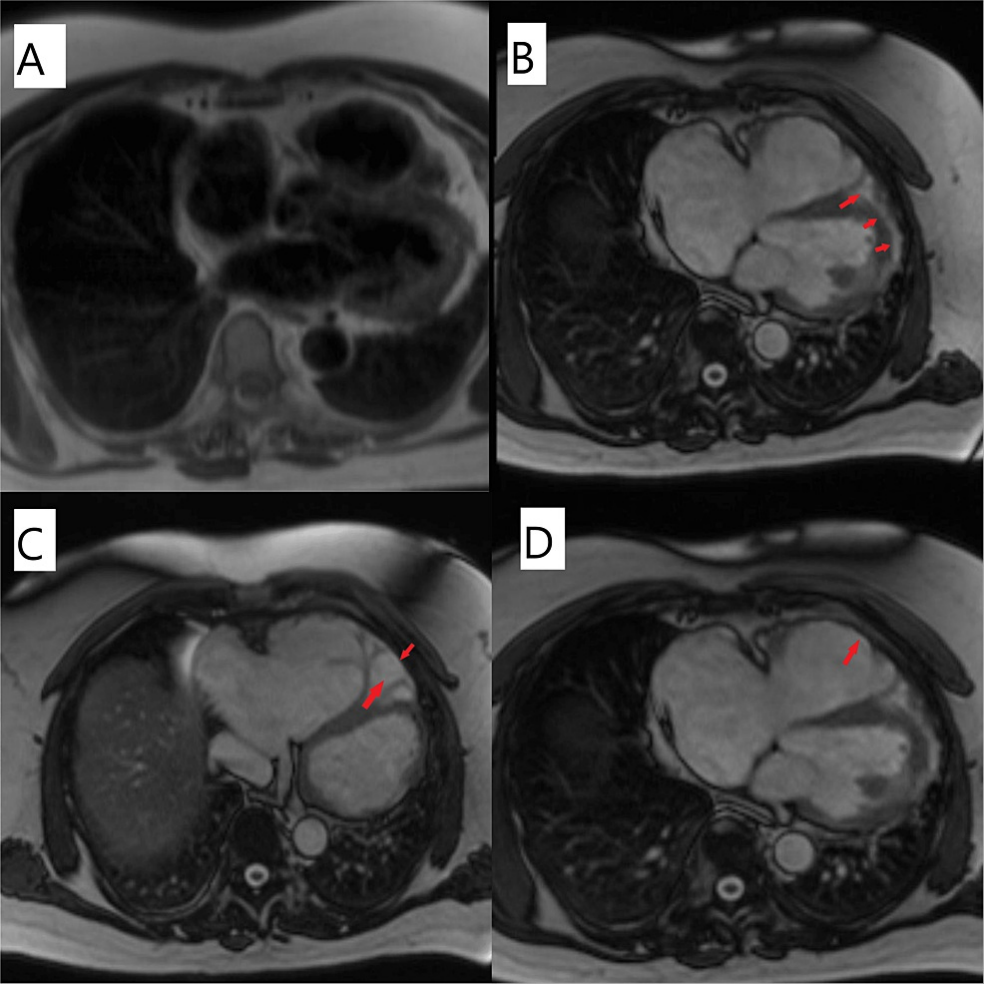
Cardiac magnetic resonance imaging (MRI) showing ARVC A - Cardiac MRI picture in diastole; B - Cardiac MRI showing the parasternal long-axis view. Red arrows show areas of inferolateral sub-epicardial left ventricular enhancement in the late Gadolinium series; C and D - Cardiac MRI showing the parasternal long-axis view. Red arrows show right ventricular global transmural fibrofatty infiltration and extensive scarring, in the late Gadolinium series. ARVC: arrhythmogenic right ventricular cardiomyopathy

Echocardiography showed a mildly impaired left ventricular (LV) systolic function with LV ejection fraction (LVEF) of 45-50% and a markedly dilated right ventricle. Much of the right ventricle was poorly visualized and there was a low probability of pulmonary hypertension, as the right ventricular systolic pressure (RVSP) was 18 mmHg. There were normal LV diastolic function parameters. He was managed with rate control medications (bisoprolol) and an antiarrhythmic agent (amiodarone). On the third day of admission, he complained of sudden-onset, left-sided hemi-paresthesia and hemiparesis. He later had an urgent stroke assessment with the CT head showing acute right posterior parietal and occipital lobe infarct, which was managed accordingly. He had a diagnostic coronary angiogram, which revealed essentially normal coronaries. Cardiac MRI revealed confirmed evidence of fatty infiltration of the right ventricle and sub-epicardial lateral LV (Figure 4). The indexed measurements are shown in Table 1 below:

**Table 1:** Cardiac MRI-indexed measurements

LEFT VENTRICULAR DIMENSIONS	RIGHT VENTRICULAR DIMENSIONS
Left Ventricular End Diastolic Volume - 98ml/m2 (62-97)	Right Ventricular End Diastolic Volume - 143ml/m2 (59-105)
Left Ventricular End Systolic Volume - 66ml/m2 (15-37)	Right Ventricular End Systolic Volume -112ml/m2 (13-42)
Left Ventricular Stroke Volume - 32ml/m2 (41-65)	Right Ventricular Stroke Volume -32ml/m2 (38-70)
Left Ventricular Ejection Fraction - 33% (58-76)	

**Figure 5:**
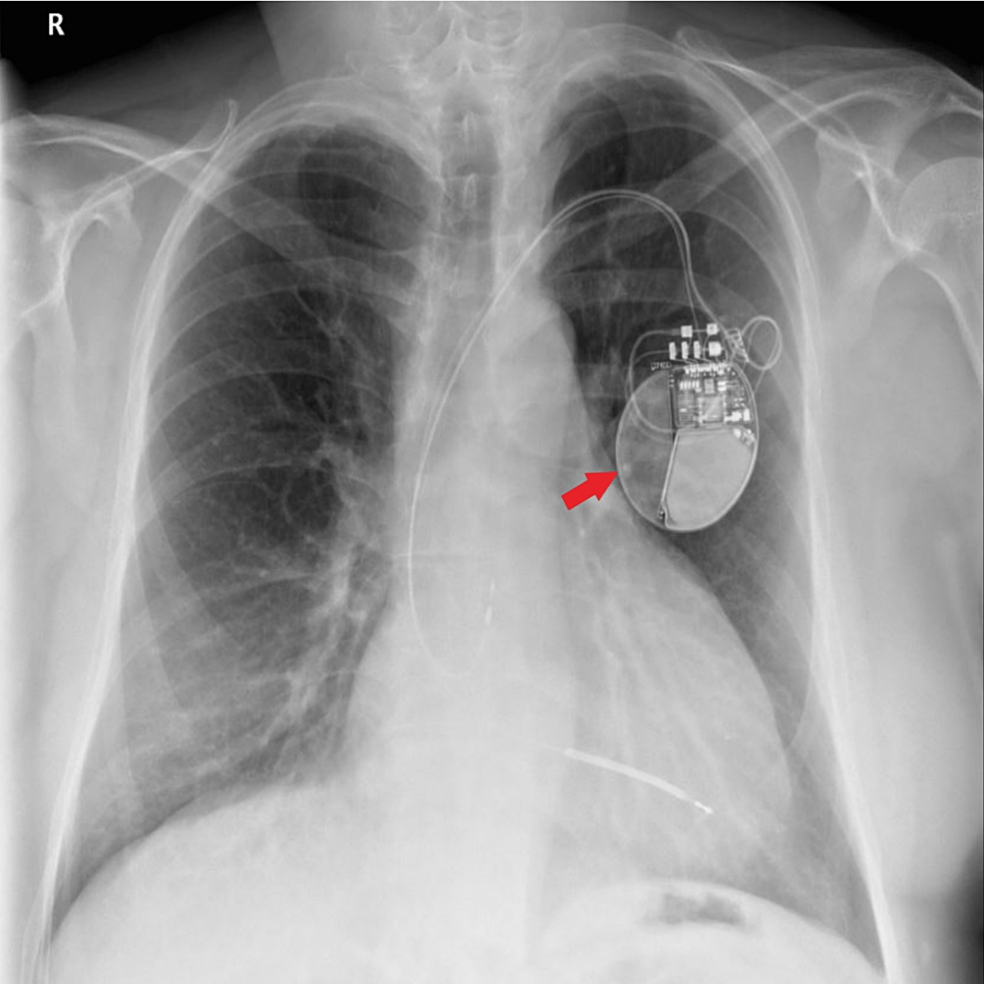
Implantable cardioverter defibrillator (ICD) in-situ Red arrow shows ICD

In summary, the cardiac MRI showed a dilated LV with severely impaired systolic function and a severely dilated LV with poor RV systolic function. There were regions of RV akinesia, dyskinesia, and microaneurysms. It also showed a global transmural RV enhancement and subepicardial enhancement in the inferior and lateral areas. Tissue characterization confirmed fatty infiltration, and the findings were in keeping with ACM with biventricular involvement and extensive scarring. He subsequently had an implantable cardioverter defibrillator (ICD) inserted (Figure 5) for secondary VT prevention, and first-degree family members were contacted for likely genetic studies and cardiovascular screening.

## Discussion

Arrhythmogenic cardiomyopathy (ACM) was previously regarded as “arrhythmogenic right ventricular dysplasia” because it predominantly affects the right ventricles; however, with a better understanding of the disease and its possible phenotypes, it was modified to be more inclusive [5,15]. It has various phenotypes, including the common right ventricular phenotype, left ventricular phenotype [3,16], and biventricular phenotype [8] as in our patient. As previously mentioned, with a general prevalence of 1:2,000-1:5,000, it is considered a rare disease based on the United Kingdom’s definition of rare disease as a condition that affects less than one in 2,000 of the population [17]. Furthermore, the true prevalence may be higher since clinical and postmortem examinations may miss the diagnosis [18]. This is further supported by the findings from various literature where ACM was responsible for up to 22% of sudden cardiac deaths in young adults [15], and even in older studies where 12 out of 60 (20%) autopsied sudden deaths in persons below 35 years of age recorded right ventricular cardiomyopathy with fatty or fibrofatty changes [19].

In the early stages of the disease, it has been described as ion channelopathies [18,20] from where it evolves to the degeneration of the cardiac muscle cells and a sequential replacement of these cells with scar tissues composed of fibrous and fatty tissues [18]. Compared to other cardiomyopathies, the pattern and location of myocardial degeneration are particularly distinctive as follows: in the right ventricles, the sub-epicardium of the free wall (especially the most stressed zones in the cardiac cycle - the posterior segment below the tricuspid annulus, the apex, and the RV outflow tract) is commonly affected in ACM compared to the involved subendocardial muscle in other cardiomyopathies [18,21]. Furthermore, in the left ventricle, myocardial deterioration and fibrosis are more noticeable in the lateral free wall's sub-epicardium and mid-myocardium [21]. Based on these structural abnormalities and the initial ion channelopathies, the victims are at risk of sudden cardiac death following arrhythmias [18,22].

ACM is a common cause of sudden cardiac death in young adults under 35 years [15] and accounts for up to 10% of fatalities from undetected cardiac illness in those over the age of 65 [23]. Our patient, in this case, is 61 years old and the mean diagnosis age of ARVD has been documented as 30 years old [8] with a range of four to 64 years [2]. Unlike in our case, most cases manifest during the fourth decade of life with other rare manifestations before puberty or in the elderly [6]. With a recorded male preponderance in a male: female ratio of 2-3:1 [2,6], our case follows this pattern. ACM was initially thought to be an Italian-based disease since most manifestations were initially described there; however, despite the paucity of data, it is now recorded across other regions [6]. No occupation has been documented as its predisposition, rather it is noted that intense exercise is probably a trigger of its “early” manifestation as sudden cardiac death and eventual post-mortem diagnoses are more common among young athletes [2,3,15,24]. Our case was a heavy-truck driver and not an athlete.

This index patient was asymptomatic, which is in keeping with the vast majority of undiagnosed ACM [8]; however, some of the initial and common presentations of the disease include lightheadedness/dizziness, syncope, and palpitations [8,12]. Palpitation is the most common symptom, manifesting in 27-67% of the cases, and occurs due to ventricular tachycardia arrhythmias [25]. Unlike our patient, a previous case report in the literature [10] described a 33-year-old male athlete who had the classical presentation of palpitation with exertional dyspnoea. Other common presentations in literature [25-27] include syncope (26-32%), sudden cardiac death (10-26%), atypical chest pain (27%), and dyspnoea (11%). Our case presented with syncope but denied chest pain, dyspnoea, and orthopnoea. Syncope is also a common but inconsistent presentation as some case reports [10,28,29] did not document it while others [30-33] did. Bariani et al. [34] described at least a single episode of chest pain in about 90% of the subjects they studied; however, this was absent in our case. Other documented findings that precede the diagnosis of ACM include heart failure [10,29,31], ventricular arrhythmias or cardiac arrest [10,30,32,33], and sudden cardiac death [10,31,33].

On its own, diabetes mellitus may predispose to cardiac arrhythmias and even structural heart disease (diabetic cardiomyopathy) [35]. This occurs commonly due to associated coronary artery disease [36]. Our patient had well-controlled diabetes and the coronary angiogram done for him was unremarkable; this suggests that the etiology of his cardiomyopathy was essentially non-ischaemic.

There is a dearth of direct studies that explores the relationship between smoking and ACMs; it is important to note that some literature [37,38] noted that smoking worsens cardiac diseases, particularly electrical conditions of the heart.

ACM being an autosomal dominant disease [12], there is a possibility of family associations for the common symptoms or sudden death clusters. Studies have shown 30-50% concordance in patients with a positive family history [39]; nonetheless, due to variable penetrance in the mode of inheritance [40], family history may be absent in certain cases. Our patient described recurrent episodes of dizziness in his son who was yet to be evaluated medically with no family history of a similar condition that he is aware of. Only one [33] of the reviewed case reports here noted a positive family history (presenting with palpitations) while others [8,10,28-30,32] did not.

**Figure 6:**
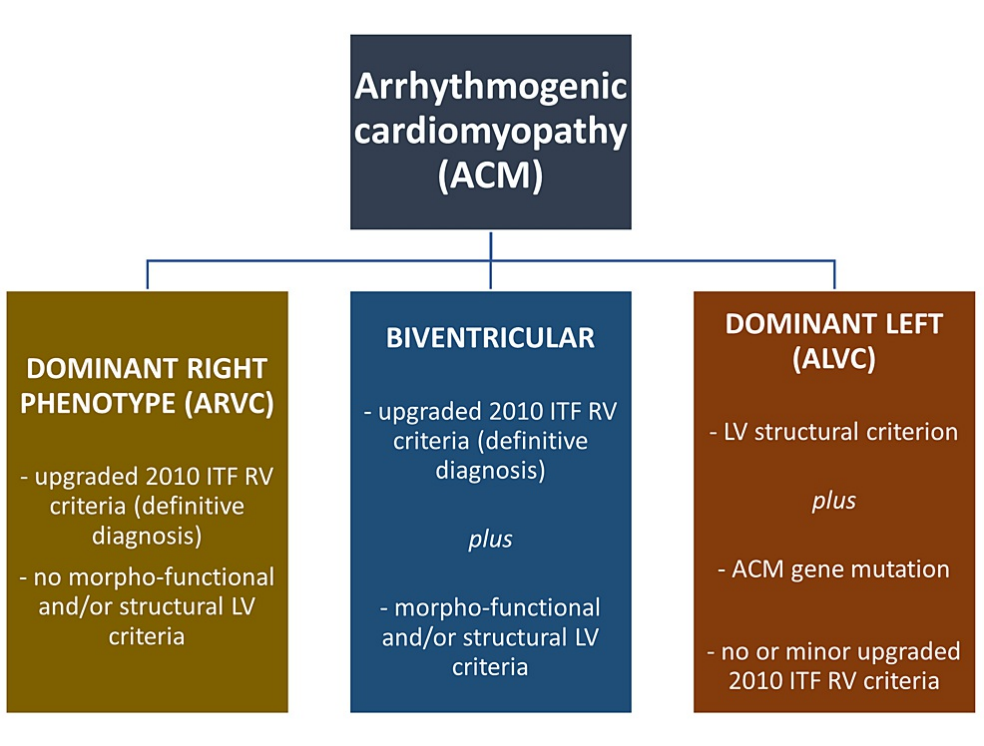
Diagnostic criteria for the various phenotypes of arrhythmogenic cardiomyopathy (ACM) Adapted from Corrado et al. (2020) [5] Attribution: NonCommercial-NoDerivatives 4.0 International (CC BY-NC-ND 4.0)

A sequel to the new nomenclature and a better understanding of the disease, newer diagnostic criteria were put forward from the revised 2010 criteria (Appendix 1) [8,41] to a more recent one - the Padua criteria (Appendix 2) [5]. This takes into cognizance, the involvement of the two ventricular compartments and six main categories viz. morpho-functional ventricular abnormalities, structural myocardial abnormalities, repolarization abnormalities, depolarization abnormalities, ventricular arrhythmias, and genetics. Though our patient did not have genetic testing, we strongly recommend early genetic testing for both the index patient and family members in the case of suspected ACM to reduce life-threatening situations since there are possible mutations that can cause ACM, for example, the DSG-2 nonsense mutation, which causes early-onset ACM [42]. The condition for the diagnosis of the various phenotypes is shown in Figure 6.

Based on the Padua criteria (Appendix 2), our patient met the major criteria for morpho-functional ventricular and structural myocardial abnormalities in both ventricles. With regard to repolarization abnormalities, he met the minor criteria of inverted T-waves in the left precordial leads (V4-V6) in the absence of complete LBBB. Depolarisation abnormalities identified terminal activation duration of QRS >55ms measured from the Nadir of the S-wave at the end of the QRS and there were no demonstrable Epsilon waves even after adopting the Fontaine lead position. Our patient also met the minor depolarization LV abnormalities of low lead QRS voltages in the absence of significant obesity, emphysema, and pericardial effusion. Our patient met the major arrhythmic Padua criteria of sustained ventricular tachycardia of LBBB morphology. He did not meet the standard case definition for family history; however, further genetic studies need to be conducted on family members as previously highlighted in this discussion.

Following the standard recommendation of ICD for secondary VT prevention [43,44], it was inserted in our patient, and he was further advised for genetic studies and cardiovascular screening of first-degree family members. Genetic studies and screening are essential for the primary prevention of sudden cardiac death and other complications following ACM, as they can be valuable in identifying up to 35%-45% of asymptomatic cases [40]. Genetic assessment is advised for first-degree relatives of individuals with ACM who have a disease-causing mutation [45] but our case has been referred for further genetic evaluation, which could not be achieved in our center due to limited facilities and logistics around the investigation.

## Conclusions

ACM is an autosomal dominant hereditary cardiopathy that is associated with a considerable risk of sudden cardiac death in young people secondary to arrhythmias. Our case report highlights the importance of doing advanced imaging studies in cases of unexplained ventricular tachycardia arrhythmias on ECG. This will help prevent mortality in suspected patients. Furthermore, it supports the reports of the occurrence of the condition in older people too, and not only in young people.

## Appendices

Appendix 1 

**Table 2:** Updated 2010 diagnostic criteria Adapted from Augusto et al. (2018) [8] RVOT - Right Ventricular Outflow Tract; PLAX - Parasternal Long Axis; PSAX - Parasternal Short Axis; BSA - Body Surface Area; RBBB - Right Bundle Branch Block; ECG - Electrocardiography; MRI - Magnetic Resonance Imaging; LBBB - Left Bundle Branch Block; ARVD - Arrhythmogenic Right Ventricular Dysplasia; AC - Arrhythmogenic Cardiomyopathy

1. Global or regional dysfunction and structural alterations	
Major	At the two-dimensional echocardiogram: - Regional right ventricular akinesis, dyskinesis or aneurysm And one of the following (end-diastolic): - RVOT PLAX ≥ 32 mm (corrected for the body surface area − PLAX/BSA ≥ 19 mm/m2) - RVOT PSAX ≥ 36 mm (corrected for the body surface area − PLAX/BSA ≥ 21 mm/m2) - Or fractional alteration of the area ≤ 33%
	At the MRI: - Regional right ventricular akinesis, dyskinesis or dys-synchrony in right ventricular contractions And one of the following: Ratio of right ventricular end-diastolic volume and BSA ≥ 110 mL/m^2^ (male) or BSA ≥ 100 mL / m^2^ (female) -- Or right ventricular ejection fraction ≤ 40%
	At the right ventricular angiography: - Right ventricular regional akinesis, dyskinesis or aneurysm
Minor	At the two-dimensional echocardiogram: - Right ventricular regional akinesis or dyskinesis And one of the following (end-diastolic): - RVOT PLAX ≥ 29 mm and < 32 mm (corrected for the body surface area − PLAX/BSA ≥ 16 mm/m^2^ and < 19 mm/m^2^) - RVOT PSAX ≥ 32 mm and < 36 mm (corrected for the body surface area − PLAX/BSA ≥ 18 mm/m^2^ and < 21 mm/m^2^) - Or fractional alteration of the area > 33% and ≤ 40%
	At the MRI: - Regional right ventricular akinesis or dyskinesis or dys-synchrony in right ventricular contraction And one of the following: - Ratio of right ventricular end-diastolic volume and BSA ≥ 100 and < 110 mL/m^2^ (man) or ≥ 90 and < 100 mL/m^2^ (woman) - Or Right ventricular ejection fraction > 40% and ≤ 45%
2. Histological characterization of the ventricular wall	
Major	- Residual myocytes <60% in the morphometric analysis (or <50% by estimation) with fibrous replacement of the right ventricular free wall in more than one sample, with or without adipose replacement in myocardial biopsy
Minor	- 60-75% of residual myocytes in the morphometric analysis (or 50-65% by estimation) with fibrous replacement of right ventricular free wall in more than one sample, with or without adipose replacement in myocardial biopsy
3. Ventricular repolarization alterations	
Major	- Inverted T waves in V1, V2 or V3 in individuals aged > 14 years in the absence of complete RBBB with QRS ≥ 120 ms
Minor	- Inverted T waves in V1 and V2 in individuals aged > 14 years in the absence of complete RBBB with QRS ≥ 120 ms or in V4, V5 or V6
	Inverted T waves in V1, V2, V3 and V4 in subjects aged > 14 years in the presence of complete RBBB with QRS ≥ 120 ms
4. Conduction/depolarization alterations	
Major	- Epsilon wave in leads V1 to V3
Minor	- Duration of QRS terminal activation ≥ 55 ms measured from the S wave nadir to the end of QRS, including R’ at V1, V2, V3 in the absence of complete RBBB of the His bundle
	- High-resolution ECG late potentials in more than one of the following three parameters in the absence of QRS ≥ 110 ms on the standard 12-lead ECG:
	- Duration of filtered QRS (fQRS) ≥ 114 ms
	- QRS terminal duration < 40 µV (low amplitude signal duration) ≥ 38 ms
	- Root mean square of the potential in the 40 ms terminals of ventricular activation (MRIs40 - mV) ≤ 20 µV
5. Arrhythmias	
Major	- Sustained or non-sustained ventricular tachycardia with complete LBBB morphology with superior axis (QRS negative or undetermined in II, III, aVF and positive in aVL)
Minor	- Sustained or non-sustained ventricular tachycardia with right ventricular outflow tract configuration, complete LBBB morphology with inferior axis (QRS positive in II, III and aVF and negative in aVL) or of indeterminate axis
	- > 500 ventricular extrasystoles in the 24-hr Holter monitoring
6. Family history	
Major	- Confirmed ARVD in a first-degree relative meeting the Task Force criteria
	- ARVD confirmed by histopathology at the autopsy or surgery in first-degree relative
	- Identification of pathogenic mutation categorized as associated or likely to be associated with ARVD* in a patient undergoing evaluation
Minor	- History of ARVD in a first-degree relative in whom it is not possible or the feasibility of confirming the presence of Task Force criteria is difficult
	- Sudden cardiac death (age < 35 years) due to suspected ARVD in first-degree relative
	- ARVD confirmed by histopathology or according to the current Task Force criteria in second-degree relative
Two major, or one major and two minor, or four minor criteria: definite diagnosis of AC. One major and one minor, or three minor criteria: borderline diagnosis; One major, or two minor criteria from different categories: possible diagnosis	

Appendix 2

**Table 3:** The Padua criteria Adapted from Corrado et al. (2020) [5] ACM = arrhythmogenic cardiomyopathy; BSA = body surface area; EDV = end diastolic volume; EF = ejection fraction; ITF = International Task Force; LBBB = left bundle-branch block; LGE = late gadolinium enhancement; LV = left ventricle; RBBB = right bundle-branch block; RV = right ventricle; RVOT = right ventricular outflow tract

CATEGORY	Right ventricle (upgraded 2010 ITF diagnostic criteria)	Left ventricle (new diagnostic criteria)
I. Morpho-functional ventricular abnormalities	By echocardiography, CMR or angiography: Major •Regional RV akinesia, dyskinesia, or bulging plus one of the following:-global RV dilatation (increase of RV EDV according to the imaging test specific nomograms)-global RV systolic dysfunction (reduction of RV EF according to the imaging test specific nomograms) Minor •Regional RV akinesia, dyskinesia or aneurysm of RV free wall	By echocardiography, CMR or angiography: Minor •Global LV systolic dysfunction (depression of LV EF or reduction of echocardiographic global longitudinal strain), with or without LV dilatation (increase of LV EDV according to the imaging test specific nomograms for age, sex, and BSA) Minor •Regional LV hypokinesia or akinesia of LV free wall, septum, or both
II. Structural myocardial abnormalities	By CE-CMR: Major •Transmural LGE (stria pattern) of≥1 RV region(s) (inlet, outlet, and apex in 2 orthogonal views) By EMB (limited indications): Major •Fibrous replacement of the myocardium in ≥1 sample, with or without fatty tissue	By CE-CMR: Major •LV LGE (stria pattern) of ≥1 Bull's Eye segment(s) (in 2 orthogonal views) of the free wall (subepicardial or midmyocardial), septum, or both (excluding septal junctional LGE)
III. Repolarization abnormalities	Major •Inverted T waves in right precordial leads (V1,V2, and V3) or beyond in individuals with complete pubertal development (in the absence of complete RBBB) Minor •Inverted T waves in leads V1 and V2 in individuals with completed pubertal development (in the absence of complete RBBB) •Inverted T waves in V1,V2,V3 and V4 in individuals with completed pubertal development in the presence of complete RBBB.	Minor •Inverted T waves in left precordial leads (V4-V6) (in the absence of complete LBBB)
IV. Depolarization abnormalities	Minor •Epsilon wave (reproducible low-amplitude signals between end of QRS complex to onset of the T wave) in the right precordial leads (V1 to V3) •Terminal activation duration of QRS ≥55 ms measured from the nadir of the S wave to the end of the QRS, including R', in V1, V2, or V3 (in the absence of complete RBBB)	Minor •Low QRS voltages (<0.5 mV peak to peak) in limb leads (in the absence of obesity, emphysema, or pericardial effusion)
V. Ventricular arrhythmias	Major •Frequent ventricular extrasystoles (>500 per 24 h), non-sustained or sustained ventricular tachycardia of LBBB morphology Minor •Frequent ventricular extrasystoles (>500 per 24 h), non-sustained or sustained ventricular tachycardia of LBBB morphology with inferior axis (“RVOT pattern”)	Minor •Frequent ventricular extrasystoles (>500 per 24 h), non-sustained or sustained ventricular tachycardia with a RBBB morphology (excluding the “fascicular pattern”)
VI. Family history/genetics	Major •ACM confirmed in a first-degree relative who meets diagnostic criteria •ACM confirmed pathologically at autopsy or surgery in a first degree relative •Identification of a pathogenic or likely pathogenetic ACM mutation in the patient under evaluation Minor •History of ACM in a first-degree relative in whom it is not possible or practical to determine whether the family member meets diagnostic criteria •Premature sudden death (<35 years of age) due to suspected ACM in a first-degree relative •ACM confirmed pathologically or by diagnostic criteria in a second-degree relative	

## References

[1] Corrado D, Basso C (2022). Arrhythmogenic left ventricular cardiomyopathy. Heart.

[2] McNally E, MacLeod H, Dellefave-Castillo L (2017). Arrhythmogenic right ventricular cardiomyopathy. GeneReviews® [Internet].

[3] Mattesi G, Cipriani A, Bauce B, Rigato I, Zorzi A, Corrado D (2021). Arrhythmogenic left ventricular cardiomyopathy: genotype-phenotype correlations and new diagnostic criteria. J Clin Med.

[4] Te Riele AS, James CA, Calkins H, Tsatsopoulou A (2021). Arrhythmogenic right ventricular cardiomyopathy in pediatric patients: an important but underrecognized clinical entity. Front Pediatr.

[5] Corrado D, Perazzolo Marra M, Zorzi A (2020). Diagnosis of arrhythmogenic cardiomyopathy: the Padua criteria. Int J Cardiol.

[6] Pilichou K, Thiene G, Bauce B (2016). Arrhythmogenic cardiomyopathy. Orphanet J Rare Dis.

[7] Corrado D, van Tintelen PJ, McKenna WJ (2020). Arrhythmogenic right ventricular cardiomyopathy: evaluation of the current diagnostic criteria and differential diagnosis. Eur Heart J.

[8] Augusto J, Abecasis J, Gil V (2018). Biventricular arrhythmogenic cardiomyopathy: a new paradigm?. Int J Cardiovasc Sci.

[9] Frank R (2018). Guy Fontaine MD PhD HDR. Eur Heart J.

[10] Latt H, Tun Aung T, Roongsritong C, Smith D (2017). A classic case of arrhythmogenic right ventricular cardiomyopathy (ARVC) and literature review. J Community Hosp Intern Med Perspect.

[11] Meyer S, van der Meer P, van Tintelen JP, van den Berg MP (2014). Sex differences in cardiomyopathies. Eur J Heart Fail.

[12] (2022). OMIM. Arrhythmogenic right ventricular dysplasia. https://www.omim.org/entry/107970.

[13] Protonotarios N, Tsatsopoulou A (2006). Naxos disease: cardiocutaneous syndrome due to cell adhesion defect. Orphanet J Rare Dis.

[14] (2022). OMIM. Naxos disease. https://www.omim.org/entry/601214.

[15] Akdis D, Brunckhorst C, Duru F, Saguner AM (2016). Arrhythmogenic cardiomyopathy: electrical and structural phenotypes. Arrhythm Electrophysiol Rev.

[16] Sen-Chowdhry S, Syrris P, Prasad SK (2008). Left-dominant arrhythmogenic cardiomyopathy: an under-recognized clinical entity. J Am Coll Cardiol.

[17] (2022). Department of Health and Social Care. The UK Rare Diseases Framework. https://www.gov.uk/government/publications/uk-rare-diseases-framework.

[18] Asimaki A, Kleber AG, Saffitz JE (2015). Pathogenesis of arrhythmogenic cardiomyopathy. Can J Cardiol.

[19] Thiene G, Nava A, Corrado D, Rossi L, Pennelli N (1988). Right ventricular cardiomyopathy and sudden death in young people. N Engl J Med.

[20] Saffitz JE, Asimaki A, Huang H (2010). Arrhythmogenic right ventricular cardiomyopathy: new insights into mechanisms of disease. Cardiovasc Pathol.

[21] Basso C, Thiene G (2005). Adipositas cordis, fatty infiltration of the right ventricle, and arrhythmogenic right ventricular cardiomyopathy. Just a matter of fat?. Cardiovasc Pathol.

[22] Basso C, Bauce B, Corrado D, Thiene G (2011). Pathophysiology of arrhythmogenic cardiomyopathy. Nat Rev Cardiol.

[23] Sen-Chowdhry S, Morgan RD, Chambers JC, McKenna WJ (2010). Arrhythmogenic cardiomyopathy: etiology, diagnosis, and treatment. Annu Rev Med.

[24] James CA, Bhonsale A, Tichnell C (2013). Exercise increases age-related penetrance and arrhythmic risk in arrhythmogenic right ventricular dysplasia/cardiomyopathy-associated desmosomal mutation carriers. J Am Coll Cardiol.

[25] Sharma GK (2022). Medscape. Arrhythmogenic right ventricular dysplasia/cardiomyopathy (ARVD/ARVC) clinical presentation. https://emedicine.medscape.com/article/163856-clinical.

[26] Dalal D, Nasir K, Bomma C (2005). Arrhythmogenic right ventricular dysplasia. A United States experience. Circulation.

[27] Hulot JS, Jouven X, Empana JP, Frank R, Fontaine G (2004). Natural history and risk stratification of arrhythmogenic right ventricular dysplasia/cardiomyopathy. Circulation.

[28] Mushtaque RS, Mushtaque R, Baloch S, Idrees M, Bhatti H (2020). Clinical manifestations and diagnostic approach to arrhythmogenic right ventricular cardiomyopathy - a case report and literature review. Cureus.

[29] Loquias M (2017). A case report on arrhythmogenic right ventricular cardiomyopathy with chronic lymphocytic leukaemia. Heart Lung Circ.

[30] Belhassen B, Shmilovich H, Nof E, Milman A (2020). A case report of arrhythmogenic ventricular cardiomyopathy presenting with sustained ventricular tachycardia arising from the right and the left ventricles before structural changes are documented. Eur Heart J Case Rep.

[31] Scarano M, Gizzi G, Mantini C (2018). Arrhythmogenic cardiomyopathy of left ventricle. A rare event, but possible. Cor Vasa.

[32] Mohamed S, Keane S, McNally C, Hayes J (2022). A case report of biventricular arrhythmogenic cardiomyopathy in a middle-aged female. Cureus.

[33] Rao U, Agarwal S, Gilbert TJ (2014). Arrhythmogenic right ventricular cardiomyopathy (ARVC): case report and review of literature. Heart Asia.

[34] Bariani R, Cipriani A, Rizzo S (2021). 'Hot phase' clinical presentation in arrhythmogenic cardiomyopathy. Europace.

[35] Tse G, Lai ET, Tse V, Yeo JM (2016). Molecular and electrophysiological mechanisms underlying cardiac arrhythmogenesis in diabetes mellitus. J Diabetes Res.

[36] Grisanti LA (2018). Diabetes and arrhythmias: pathophysiology, mechanisms and therapeutic outcomes. Front Physiol.

[37] Kayali S, Demir F (2017). The effects of cigarette smoking on ventricular repolarization in adolescents. Einstein (Sao Paulo).

[38] Park J, Lee HJ, Kim SK (2018). Smoking aggravates ventricular arrhythmic events in non-ischemic dilated cardiomyopathy associated with a late gadolinium enhancement in cardiac MRI. Sci Rep.

[39] (2022). Johns Hopkins Medicine. Genetics of arrhythmogenic right ventricular dysplasia / cardiomyopathy (Arvdc). https://www.hopkinsmedicine.org/health/conditions-and-diseases/genetics-of-arrhythmogenic-right-ventricular-dysplasia-cardiomyopathy-arvdc.

[40] Quarta G, Muir A, Pantazis A (2011). Familial evaluation in arrhythmogenic right ventricular cardiomyopathy: impact of genetics and revised task force criteria. Circulation.

[41] Marcus FI, McKenna WJ, Sherrill D (2010). Diagnosis of arrhythmogenic right ventricular cardiomyopathy/dysplasia: proposed modification of the task force criteria. Circulation.

[42] Shiba M, Higo S, Kondo T (2021). Phenotypic recapitulation and correction of desmoglein-2-deficient cardiomyopathy using human-induced pluripotent stem cell-derived cardiomyocytes. Hum Mol Genet.

[43] Epstein AE, DiMarco JP, Ellenbogen KA (2008). ACC/AHA/HRS 2008 guidelines for device-based therapy of cardiac rhythm abnormalities: a report of the American College of Cardiology/American Heart Association Task Force on practice guidelines (writing committee to revise the ACC/AHA/NASPE 2002 guideline update for implantation of cardiac pacemakers and antiarrhythmia devices) developed in collaboration with the American Association for Thoracic Surgery and Society of Thoracic Surgeons. J Am Coll Cardiol.

[44] Calkins H (2015). Arrhythmogenic right ventricular dysplasia/cardiomyopathy - three decades of progress. Circ J.

[45] Groeneweg JA, Bhonsale A, James CA (2015). Clinical presentation, long-term follow-up, and outcomes of 1001 arrhythmogenic right ventricular dysplasia/cardiomyopathy patients and family members. Circ Cardiovasc Genet.

